# Protective antibody threshold of RTS,S/AS01 malaria vaccine correlates antigen and adjuvant dose in mouse model

**DOI:** 10.1038/s41541-023-00714-x

**Published:** 2023-08-10

**Authors:** Christopher J. Genito, Katherine Brooks, Alexis Smith, Emma Ryan, Kim Soto, Yuanzhang Li, Lucile Warter, Sheetij Dutta

**Affiliations:** 1 Structural Vaccinology Laboratory, Biologics Research and Development Branch, Walter Reed Army Institute of Research, Silver Spring, MD 20910 USA; 2 Center for Enabling Capabilities, Walter Reed Army Institute of Research, Silver Spring, MD 20910 USA; 3 GSK, Rixensart, Belgium

**Keywords:** Malaria, Adjuvants

## Abstract

Mouse models are useful for the early down-selection of malaria vaccine candidates. The Walter Reed Army Institute of Research has optimized a transgenic *Plasmodium berghei* sporozoite challenge model to compare the efficacy of *Plasmodium falciparum* circumsporozoite protein (CSP) vaccines. GSK’s RTS,S vaccine formulated in the adjuvant AS01 can protect malaria-naïve individuals against malaria. We report that the RTS,S/AS01 vaccine induces high level sterile protection in our mouse model. Down titration of the antigen at a constant AS01 dose revealed a potent antigen dose-sparing effect and the superiority of RTS,S/AS01 over a soluble CSP antigen. RTS,S-mediated protective immunity was associated with a threshold of major repeat antibody titer. Combined titration of the antigen and adjuvant showed that reducing the adjuvant could improve antibody boosting post-3rd vaccination and reduce the threshold antibody concentration required for protection. Mouse models can provide a pathway for preclinical assessment of strategies to improve CSP vaccines against malaria.

## Introduction

The World Health Organization (WHO) estimated 241 million cases of malaria resulting in 627,000 deaths during 2020^1^. Despite the success of control programs, malaria appears to be on the rise, and the availability of an effective vaccine can greatly accelerate its elimination^2^. *Plasmodium (P.) falciparum* circumsporozoite protein (CSP) is the most abundant protein on the sporozoite stage, and is believed to be essential for structural integrity, motility, and invasion of human hepatocytes^3^. Structurally, *P. falciparum* CSP consists of a conserved N-terminal domain, 38 NANP major repeats, 4 NVDP repeats, and a polymorphic C-terminal domain. Antibodies against CSP can block hepatocyte infection by sporozoites by forming a precipitate on the sporozoite surface^4^. Vaccination with CSP elicits sterilizing protection against controlled human malaria infection (CHMI) delivered via mosquito bite^5^.

RTS,S/AS01_E_ (Mosquirix) is a first-generation licensed malaria vaccine. Following a 2021 WHO recommendation for routine use in children ≥5 months of age living in areas with moderate to high malaria transmission^6^, RTS,S is undergoing pilot implementation in Ghana, Kenya, and Malawi. RTS,S is a mixed particle containing the hepatitis B *S* antigen fused to 18 copies of the NANP major repeats and the C-terminal domain of *P. falciparum* 3D7 strain CSP, along with free hepatitis B *S* antigen^7^. RTS,S is formulated with GSK’s proprietary adjuvant AS01_E_, which contains a liposomal formulation of two immuno-stimulants: the toll-like receptor 4 agonist (Monophosphoryl Lipid A (MPL)^8^ and QS-21 saponin isolated from the bark of the *Quillaja saponaria* tree^9^ (25 μg MPL and 25 µg QS-21). The adjuvant AS01 mediates immune enhancement via the synergistic action of MPL and QS21^10^. In the early 1990s, the Walter Reed Army Institute of Research (WRAIR) collaborated with GSK and conducted homologous CHMI trials of RTS,S formulations in US volunteers^11^. Randomized CHMI studies showed efficacy in the 30–50% range when using RTS,S with AS02 (an adjuvant system containing 50 μg MPL and 50 μg QS-21 in an oil-in-water emulsion) or AS01_B_ (an adjuvant system containing 50 μg MPL and 50 µg QS-21 in a liposomal formulation)^12^. The efficacy of RTS,S/AS01_B_ in Kenyan adults against malaria was subsequently reported to be ~30% over a 12-month period^13^. In 1–4-year-old Mozambiquian children, 3 doses of RTS,S formulated together with AS02 showed ~30% efficacy over 42 months^14,15^. A pediatric formulation of RTS,S/AS01_E_ administered to 5–17-month-old children in Kenya and Tanzania showed 39% and 46% efficacy at 12 or 15 months post-vaccination^16^. In a multicentric Phase 3 trial, the efficacy of RTS,S/AS01_E_ was reported as 55% against clinical disease and 47% against severe disease over the first 12 months of follow-up post-3rd vaccination^17^. Pivotal studies in Africa have reported 46% efficacy against clinical disease and 36% efficacy against severe disease over the first 18 months of follow-up post-3rd vaccination^18^. In a 4-year follow-up in children that received 4 vaccinations of RTS,S/AS01_E_, 36% efficacy against clinical disease and 32% efficacy against severe disease was reported^19^. These clinical trials indicated that RTS,S vaccination in its current formulation and schedule exhibits lower efficacy in malaria-endemic areas than in CHMI^20^.

There are several lines of evidence to suggest that RTS,S vaccine efficacy can be further improved by optimizing the vaccine regimen. Delaying and fractionating the 3rd dose of RTS,S formulated in either AS01 or AS02 (DFD regimen) was shown to increase protection in a CHMI trial^21,22^. Reduced booster dose regimens of RTS,S/AS01_E_ also showed promising efficacy when the trial participants underwent challenge 3 months after the last immunization^23^. While the DFD regimen showed promising improvement in efficacy in naïve adults, it performed comparably to the standard regimen in pediatric populations living in malaria endemic regions^24^. This may due to the exposure of RTS,S vaccinees in the field to the parasite at the time of booster vaccination and during the 20 months of efficacy follow-up. In another unrelated study, adenovirus priming followed by 2 doses of RTS,S/AS01 showed protection at a lower antibody titer compared to the standard RTS,S regimen^25^. It remains unclear if this effect was due to improved antibody quality or an augmented T-cell response elicited by the prime-boost regimen. Further studies with RTS,S/AS01 formulation and schedule are needed to determine if antibody quality and vaccine efficacy can be improved in endemic areas.

Several models have been explored to evaluate the efficacy of *P. falciparum* CSP-based vaccines. While in vitro assays for sporozoite traversal and motility are available^26^, hepatocyte invasion inhibition assays are difficult to standardize due to quality variations in primary hepatic cells^27^ and mosquito-derived sporozoites and the lack of the three-dimensional microenvironment of human liver. Wild-type mice are not susceptible to *P. falciparum* sporozoites and immunodeficient mouse strains reconstituted with human liver cells are required^28,29^. Reconstituted mouse model experiments can be expensive and restricted to small group sizes. Rodent *Plasmodium* species engineered to express a functional *P. falciparum* CSP gene^30,31^ can reliably infect common inbred strains of mice and are now routinely used for early screening of CSP vaccine candidates^32–40^. The R21/Matrix-M^5,41^, the FL-CSP/GLA-LSQ^42^, FMP013/ALFQ vaccines^40^, and the monoclonal antibodies CIS43LS and L9^43,44^ were all transitioned to the clinic using transgenic *P. berghei* challenge data in mice. The WRAIR malaria program has previously standardized a transgenic mouse challenge model for evaluating CSP vaccines^39^. The model uses *P. berghei* parasites where the endogenous CSP gene is replaced by the full-length *P. falciparum* CSP^38,39^. As WRAIR continues to evaluate ways to improve the efficacy of RTS,S, assessing the clinically successful RTS,S/AS01 vaccine in our mouse challenge model has been of considerable interest. Here, we report immunogenicity and efficacy studies of RTS,S/AS01 in the WRAIR mouse challenge model. The dose of antigen and the vaccine formulation were titrated to determine the 50% protective dose (PD_50_). Our findings show associations between antigen dose, adjuvant dose, antibody concentration, and protection in the mouse model.

## Results

### Pilot study

**Fig. 1:**
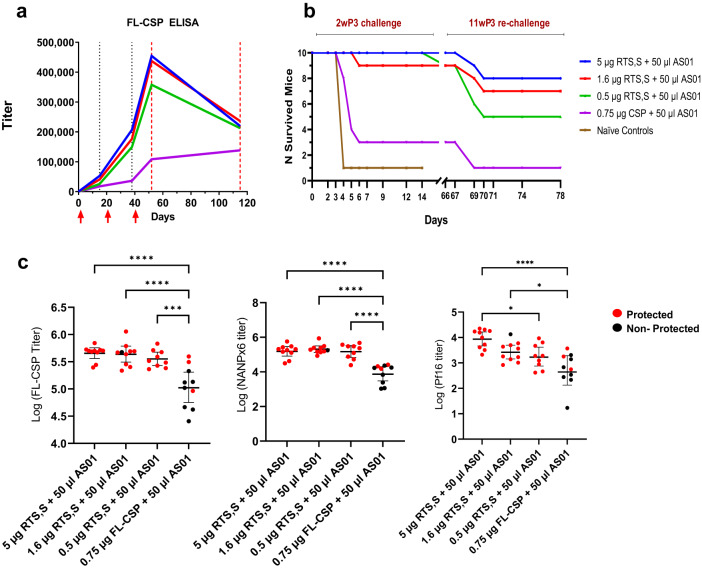
Efficacy and immunogenicity data for the pilot experiment. **a** Three vaccinations of RTS,S or FL-CSP (red arrows) were administered at 3-week intervals and bleeds were collected on day 21 (3 weeks post-1st vaccine), day 42 (3 weeks post-2nd vaccine), and day 52 (2 weeks post-3rd vaccine) (dotted black lines). Parasite challenge (red dotted lines) was performed at day 52 (2 weeks post-3rd vaccine), and surviving mice were re-challenged on day 115 (11 weeks post-3rd vaccine). Geometric mean titers (GMT) against FL-CSP are plotted. **b** Survival curves for 2 weeks post-3rd vaccine challenge (day 0–14 post-challenge) and 11 weeks post-3rd vaccine re-challenge (days 67–78). All naïve controls for the re-challenge were infected by day 5 (*not plotted*). **c** Log titers at 2 weeks post-3rd vaccine for individual mice against FL-CSP (left), NANPx6 (middle), and Pf16 peptide (right). Protected mice are shown in red and non-protected in black. Error bars represent geometric mean ± 95% CI and significant ANOVA *P* values corrected for multiple comparisons (*<0.05; ***<0.001; ****<0.0001).

C57Bl/6 mice (*n* = 10) were immunized with either a 3-fold titration of the RTS,S antigen (5 µg, 1.6 µg, or 0.5 µg) in 50 µL AS01 adjuvant or 0.75 µg of a nearly full-length soluble CSP (FL-CSP) formulated in 50 µL AS01^40^. The FL-CSP dose contained an equivalent molar amount of CSP as the 5 µg RTS,S group. AS01 used in this study contained 100 μg/mL MPL and 100 µg/mL QS-21 in a liposome-based formulation; each vaccine dose therefore contained 5 μg MPL and 5 μg QS-21. Mice achieving sterile protection from the initial challenge at 2 weeks post-3rd vaccine were re-challenged at 11 weeks post-3rd vaccine. Titers increased following the 2nd and 3rd vaccination, but dropped by ~50% in the 9 weeks that followed the 3rd vaccination (Fig. 1a). At 2 weeks post 3rd vaccine, ≥90% protection across all RTS,S groups was observed (Fig. 1b). The FL-CSP and major repeat (NANPx6) antibody titers at the 2 week post-3rd vaccine time point did not differ significantly between 5 and 0.5 μg RTS,S, although the C-terminal (Pf16) titers were significantly lower at 0.5 μg (Fig. 1c). Antibody titers elicited by the FL-CSP/AS01 vaccine were lower than the 5 µg RTS,S group and resulted in only 30% protection (RTS,S groups vs. FL-CSP, Fisher’s exact test, *P* value < 0.01). Rechallenge showed a drop in protection levels, e.g., 50% protection at 0.5 μg, and 80% at 5 μg RTS,S dose. This pilot study showed that when the AS01 dose was kept constant, saturating anti-CSP titers and high level protection were elicited between 5 and 0.5 µg RTS,S dose. The superiority of RTS,S/AS01 over an equivalent dose of soluble FL-CSP/AS01 was also established.

### Antigen titration

**Fig. 2:**
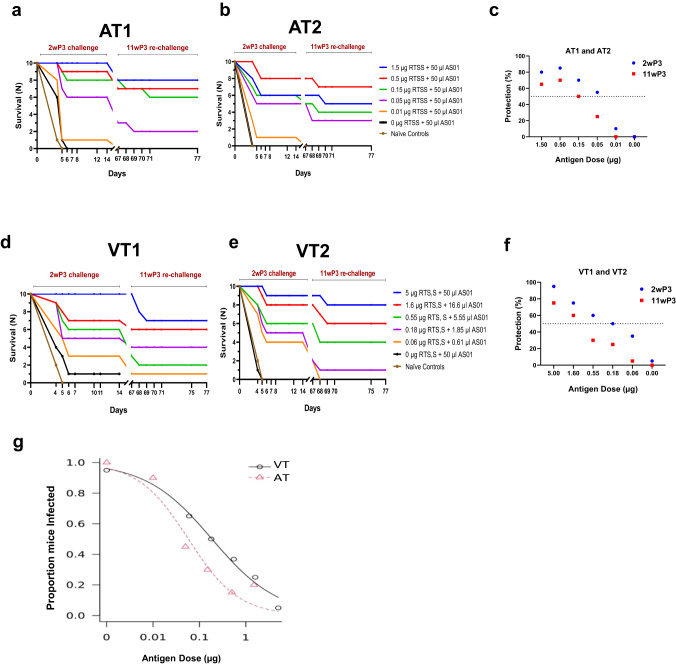
Efficacy data for antigen titration (AT) and vaccine titration (VT). Survival curves 2 weeks post-3rd vaccine challenge and 11 weeks post-3rd vaccine re-challenge for the (**a**) AT1, (**b**) AT2, (**d**) VT1, and (**e**) VT2 experiments. All naïve controls for the re-challenge were infected by day 5 (not plotted). **c**, **f** Mean percent sterile protection for 2 weeks post-3rd vaccine and 11 weeks post-3rd vaccine challenge (2wP3, 11wP3) for AT and VT experiments, respectively (*n* = 20 per group). The dotted black line denotes 50% protection. **g** Estimation of PD_50_ using a two parameter log logistic model. The fitted dose-response curves for AT and VT are shown.

To examine the effect of RTS,S antigen titration, two identical and independent challenge experiments, AT1 and AT2, were conducted and the 50% protective antigen dose (PD_50_) was determined. C57Bl/6 mice (*n* = 10 per group in each study) received 3-fold dilutions of RTS,S antigen (1.5 µg to 0.01 µg) in a constant volume of 50 µL AS01 (Table 1, Fig. 2a–c). For both AT1 and AT2, a sporozoite challenge at 2 week-post 3rd vaccination showed a 1–2 day delay in patency or sterile protection in most RTS,S groups. Combining protection outcomes from AT1 and AT2 (*n* = 20 per group) showed that 3 vaccinations with 1.5 to 0.05 µg RTS,S in 50 µL AS01 elicited significant protection compared to the naïve control group (Fisher’s exact test, *P* values < 0.004). A log logistic model estimated the 50% protective dose of the RTS,S antigen to be 0.058 µg in 50 µL AS01 for the two antigen titration (AT) experiments (SE = 0.016; 95% CI = 0.026**–**0.09) (Fig. 2g). Rechallenge of surviving mice showed a drop in efficacy across all groups, but protection remained statistically significant compared to the naïve controls (Fisher’s exact test, *P* values < 0.04). Together with the pilot study, the AT1 and AT2 experiments indicated that very low doses of RTS,S antigen in 50 µL AS01 can confer sterile protection in the rodent model.

**Table 1:** Experimental groups.

Study name	Vaccine/outcome	Groups						
Pilot (*n* = 10 per group)	RTS,S (µg)	5	1.6	0.5	–	–	–	–
	AS01 (µl)	50	50	50	–	–	–	–
	Protection (%)	100	90	90	–	–	–	–
AT1 and AT2 (*n* = 10 per group)	RTS,S (µg)	–	1.5	0.5	0.15	0.05	0.01	0
	AS01 (µl)	–	50	50	50	50	50	50
	Protection (%)	–	80	85	70	55	10	0
VT1 and VT2 (*n* = 10 per group)	RTS,S (µg)	5	1.6	0.55	0.18	0.06	–	0
	AS01 (µl)	50	16.6	5.55	1.85	0.61	–	50
	Protection (%)	95	75	60	50	35	–	5

Mice (*n* = 10 per group) received three immunizations at 3-week intervals. The pilot study also included a group of mice that received 0.75 µg FL-CSP as the control. Naïve mice were used as infectivity controls during sporozoite challenge.

### Vaccine titration

In two identical and independent challenge experiments, designated VT1 and VT2, the vaccine (antigen + adjuvant) was titrated in 3-fold increments. Antigen doses in vaccine titration (VT) varied from 5 µg RTS,S + 50 µL AS01 down to 0.06 µg RTS,S + 0.61 µL AS01 and encompassed similar antigen doses tested in AT at lower adjuvant volumes (Table 1). Challenge showed a 1–2 day delay in patency or sterile protection across VT1 and VT2 groups (Fig. 2d, e). While protection in the AT study saturated above 0.5 µg in 50 µL adjuvant dose (Fig. 2c), VT protection curves revealed a dose effect with a gradual reduction in protection between 5 and 0.55 µg (Fig. 2f). Sterile protection for VT groups was significant compared to the naïve controls (Fisher’s exact test, *P* values < 0.04). Furthermore, sterile protection at 5 µg RTS,S + 50 µL of AS01 was significantly better than 0.55 µg + 5.5 µL (Fisher’s test *P* value = 0.02), 0.18 µg +1.85 µL (*P* value = 0.003) and 0.06 µg + 0.61 µL groups (*P* value = 0.0001). Likewise, sterile protection at 1.6 µg RTS,S + 16.6 µL AS01 was significantly better than at 0.06 µg + 0.61 µL group (Fisher’s exact test, *P* value < 0.03). Rechallenge of surviving mice resulted in infection in some RTS,S group mice, but overall protection levels remained significant compared to the control group (Fisher’s test *P* values < 0.04). The 50% protective dose estimate for the two VT experiments was 0.186 µg RTS,S antigen in ~1.85 μl AS01 (SE = 0.065; 95% CI = 0.058**–**0.315) (Fig. 2g). Overall, the VT study protection outcomes titrated better with the antigen dose, requiring 3.2-fold higher antigen to achieve 50% protection, as compared to AT.

### Antibody boosting patterns in AT vs. VT

**Fig. 3:**
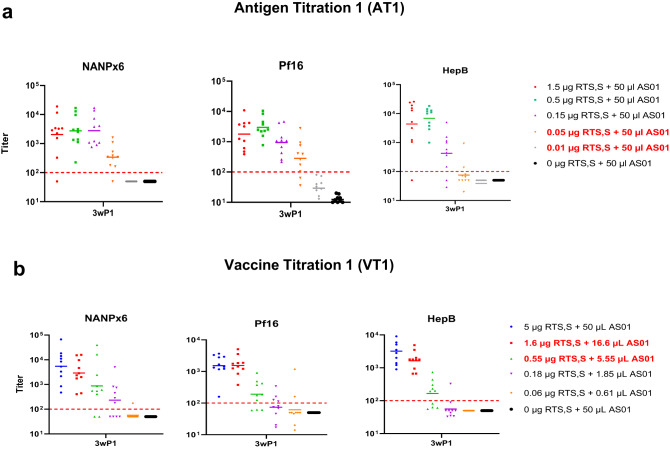
Seroconversion post-1st vaccination. Geometric mean titer (GMT ± 95% CI) for NANPx6, Pf16, and HepB ELISA titers were plotted at 3 weeks post-1st vaccine for (**a**) AT1 and (**b**) VT1. The dashed red line denotes the OD = 1 titer required for seroconversion. The lowest RTS,S dose that achieved ≥80% seroconversion in AT1 and VT1 groups after the 1st vaccination were annotated in red.

**Fig. 4:**
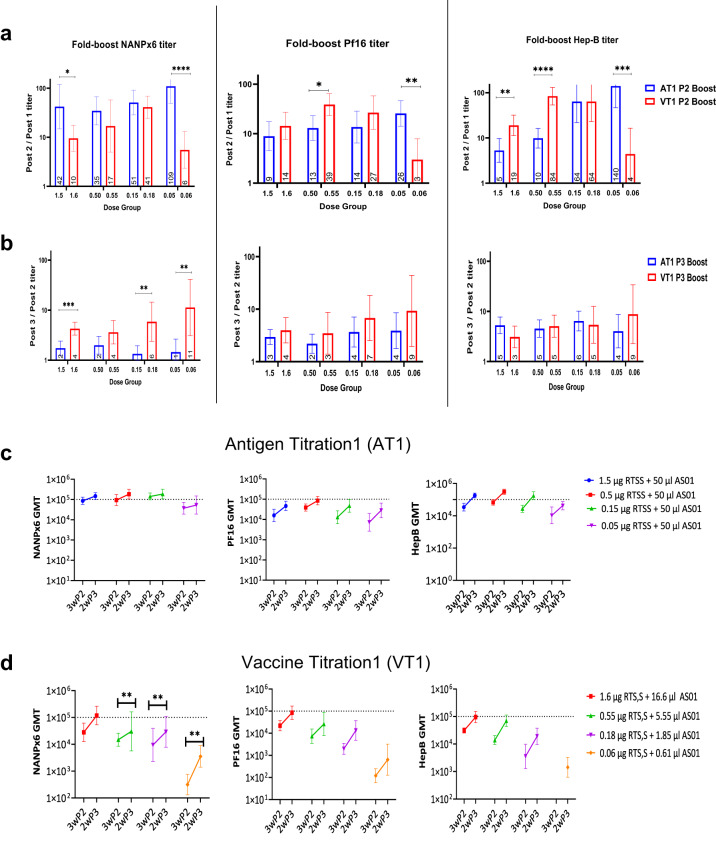
Boosting patterns for AT1 vs. VT1. Fold GMT boost in antibody titers for AT1 and VT1 experiments observed after (**a**) the 2nd vaccination (ratio of post-2nd/post-1st vaccine titer) or (**b**) the 3rd vaccination (ratio of post-3rd/post-2nd titer). Magnitude of antibody titers at 3 weeks post-2nd and 2 weeks post-3rd vaccine for (**c**) AT1 and (**d**) VT1 against NANPx6, Pf16, and HepB antigens. Dotted lines represent saturating OD415 = 1 titer of 105. Significant *P* values for Mann–Whitey *U* tests are indicated. * *P* < 0.05; ** <0.01; ***<0.001; ****<0.0001). Error bars represent geometric mean and 95% CI.

To determine the effect of different titrations on priming and boosting of antibody responses, antibody acquisition patterns were longitudinally analyzed for seroconversion post-1st vaccination (Fig. 3) and for fold-change in titer after the 2nd and 3rd vaccinations (Fig. 4). An approximately 10-fold lower antigen dose was required to seroconvert ≥80% mice after the 1st vaccination (ELISA titer > 100) in AT1, as compared to VT1 (Fig. 3), indicating an antigen dose-sparing effect of higher AS01 doses in AT1 after the priming vaccination. For booster doses, the fold-change of NANPx6, Pf16 and Hep-B titers post-2nd vaccination (Fig. 4a) was higher in magnitude than post-3rd vaccination (Fig. 4b). The dose-sparing effect of higher AS01 in AT1 on ELISA titers was most obvious after the 2nd vaccination and at the lowest antigen dose (AT 0.05 µg RTS,S + 50 µL AS01 *vs*. VT 0.06 µg RTS,S + 0.61 µL AS01, Mann-Whitney test *P* values < 0.01) (Fig. 4a). However, this dose-sparing effect was largely absent after the 3rd dose, despite the higher AS01 doses in AT (Fig. 4b). The NANPx6 ELISA titers, which historically have been correlated with RTS,S-mediated protection^45^, showed greater boosting post-3rd vaccination for VT1 groups than AT1 (Fig. 4b), contrary to the NANPx6 boosting trends observed post-2nd vaccination (Fig. 4a). The geometric mean titer (GMT) against NANPx6 in AT1 was considered saturating (OD = 1 titers ~10^5^) across antigen doses after the 2nd vaccination (Fig. 4c), as no significant boosting was observed when comparing post-2nd and post-3rd vaccination titers (Mann-Whitney test *P* values not significant). In contrast, the NANPx6 GMT in VT1 showed significant boosting between 2nd and 3rd vaccinations (Mann–Whitney test, *P* values < 0.01), and an effect of antigen dose titration on titers was clearly observed (Fig. 4d). Together, higher adjuvant doses in AT1 regimens improved the major repeat antibody boosting post 2nd vaccination, but it may have masked a critical antigen dose effect and inhibited the major repeat titer boosting post 3rd vaccination.

### Relationship between vaccine dose and immunogenicity

**Fig. 5:**
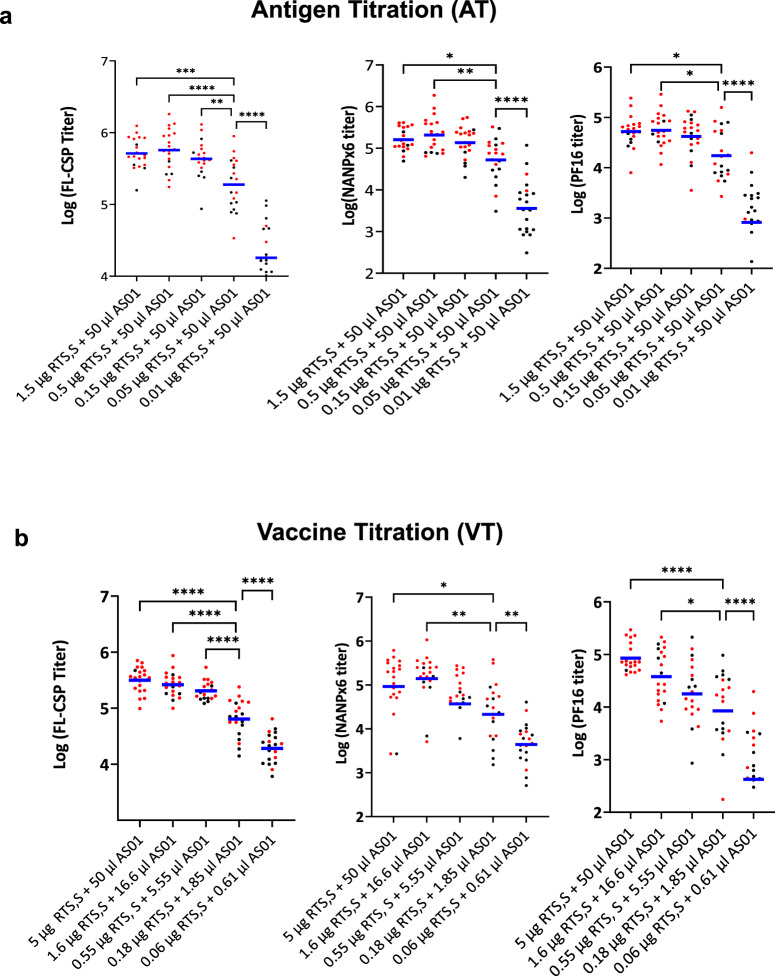
Vaccine dose and antibody titer. Log GMT 2 weeks post-3rd vaccine titers plotted for (**a**) AT and (**b**) VT experiments against FL-CSP, NANPx6, and Pf16 plate antigens. Protected mice are shown in red and non-protected in black. Significant ANOVA *P* values corrected for multiple comparisons *P < 0.05; **<0.01; ***<0.001; ****<0.0001).

We analyzed the effect of the AT and VT regimens on ELISA titers and challenge outcomes at 2 weeks post-3rd vaccination (Fig. 5a, b; red = protected and black = non-protected). In AT (*n* = 20 per group), over a 10-fold change in antigen dose (1.5 to 0.15 µg) in 50 µL AS01 resulted in no significant drop in titer and a small drop in protection (85 to 70%, Table 1). In VT, a similar antigen dose range (1.6–0.18 µg) in lower 1.85 to 16.6 µL AS01 volumes showed a linear (*R*^2^ = 0.98) and statistically significant drop in titer accompanied by drop in protection from 75% to 50% (Table 1). Hence, the higher adjuvant used in AT regimens saturated the immunogenicity of RTS,S at higher antigen doses in the mouse model. At a relatively high antigen dose (1.5 µg and 1.6 µg), a 3-fold higher adjuvant in AT (50 µL vs. 16.6 µL AS01) resulted in similar titers; but at lower antigen dose (0.05 µg vs. 0.06 µg), an 80-fold higher adjuvant in AT regimen (50 µL vs. 0.61 µL AS01) resulted in a 10- to 30-fold higher titer favoring AT (Supplementary Fig. 1). Therefore, the antigen dose sparing effect of AS01 on RTS,S immunogenicity was most significant when the antigen dose was limiting.

### Relationship between immunogenicity and protection

**Fig. 6:**
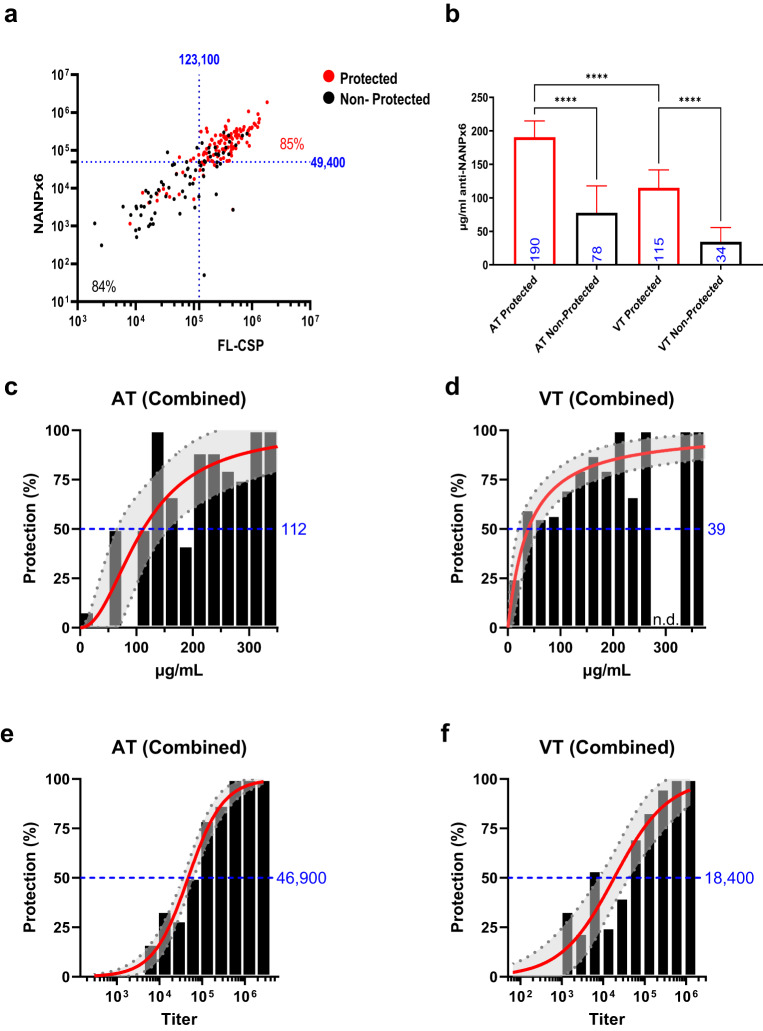
Models of vaccine protective efficacy. **a** NANPx6 vs. FL-CSP titers for combined AT and VT studies (*n* = 280) and optimal protection titer cutoffs predicted by AUC analysis are shown as dotted lines. The percentage of protected (red) or non-protected (black) mice are shown in high and low titer quadrants. **b** Geometric mean (±95% CI) anti-NANPx6 antibody concentration (µg/ml) determined by BLI for protected and non-protected AT and VT experiment mice; ****ANOVA *P* value < 0.0001. **c**, **d** Modeling of antibody protective efficacy using Hill equation for µg/ml concentrations or (**e**, **f**) NANPx6 ELISA titer following challenge. Histogram shows the distribution of antibody concentrations. The shaded regions represent the 95% CI for estimated vaccine efficacy. Antibody amounts predicted for 50% efficacy are shown in blue.

To further investigate the relationship between antibody titers and protection, a receiver operator characteristic (ROC) curve was plotted using the 2 weeks post-3rd vaccine titers for AT1, AT2, VT1, and VT2 (*n* = 280) (Supplementary Figure 2; Table 2). The area under a ROC curve (AUC) equaling 0.5 suggests random classification, while AUC = 1.0 indicates a perfect test accuracy^46^. The AUC values for NANPx6 titer (0.91) and FL-CSP titer (0.90) were indicative of excellent, non-random classification of protection. In contrast, Pf16 (AUC = 0.86) was only slightly less predictive of protection (NANPx6 vs. Pf16 AUC values, Chi-square test, *P* value = 0.007). The optimal titer cutoffs predicted by the ROC analysis on combined AT and VT experiments showed that NANPx6 titer (cutoff = 49,400) and FL-CSP titer (cutoff = 123,100) correctly classified 85% of protected and 84% of non-protected outcomes (Fig. 6a). The ROC analysis also revealed that FL-CSP, NANPx,6 and Pf16 protective titer cut-offs for AT were higher than VT experiments (Table 2).

**Table 2:** ROC predicted optimal cutoffs and area under the curve (AUC).

Predictor	Cutoff	AUC
AT1, AT2, VT1, VT2		
FL-CSP	123100	0.9047
NANPx6	49400	0.9124
PF16	8882	0.8638
AT1, 2		
FL-CSP	266300	0.939
NANPx6	49400	0.9271
PF16	10958	0.8967
VT1, 2		
FL-CSP	79100	0.8886
NANPx6	14600	0.9018
PF16	4957	0.8378
µg/ml anti-NPNAx6		
AT1	204	0.7186
AT2	179	0.7348
VT1	122	0.8704
VT2	79	0.8239

Titers at 2-week post-3rd vaccination against FL-CSP, NANPx6, and Pf16 plate antigen and µg/ml anti-NPNAx6 concentration determined by BLI for AT1,2 and VT1,2 experiments, analyzed separately or combined.

Given the difference in ROC cutoffs between AT and VT, an avidity assay was conducted to detect any differences in avidity. The AT (1.5 µg RTS,S + 50 µL AS01) and VT (1.6 µg RTS,S + 1.85 µL AS01) had similar ELISA titers and showed no significant difference in avidity (Supplementary Fig. 3a). Likewise, AT1 (0.05 µg RTS,S + 50 µL AS01) and VT1 (0.18 µg RTS,S + 1.86 µL AS01) groups that had similar protection ~50% showed no significant difference in avidity (Supplementary Fig. 3b). Overall, the magnitude of antibodies was highly predictive of protection and higher titers were required for protection in the higher adjuvant AT regimen compared to VT.

### Protective antibody concentrations

Since NANPx6 titer was associated with protection outcome, the concentration of major repeat antibodies required for protection was determined using a label-free biolayer interferometry assay (BLI). The geometric mean concentration of NANPx6 antibodies in protected mice (190 µg/mL in AT and 115 µg/mL in VT) were higher than in non-protected mice (78 µg/mL and 34 µg/mL, respectively) and, importantly, the protected AT mice had significantly higher antibody concentration than the protected VT mice (Fig. 6b). An ROC analysis on AT vs. VT major repeat antibody concentrations revealed that the protective cut-offs for AT1 and AT2 (204 and 179 µg/mL, respectively) were higher than those of VT1 and VT2 (122 and 79 µg/mL, respectively) (Supplementary Fig. 4 and Table 2). Since the ROC method assumes a threshold protective titer exists, we also modeled efficacy over incremental increases in major repeat antibody concentration (Fig. 6c–f). Protection vs. NANPx6 µg/mL or titer showed a good fit to the Hill equation (R^2^ range 0.69**–**0.98), further confirming correlation of protection with major repeat antibodies. The µg/ml and NANPx6 titers associated with 50% efficacy for AT (112 µg/mL and 46,900, respectively) were higher than for VT (39 µg/mL and 18,400, respectively) confirming that protective antibody threshold in VT were lower than AT regimens.

## Discussion

Multiple key observations of RTS,S/AS01 vaccination studies in humans were recapitulated in the present mouse model. Major repeat-binding antibody titers in RTS,S human trials have been associated with protection^47,48^, and the level of protection drops as repeat titers wane^12^. In mice, RTS,S/AS01 elicited a strong sterilizing protection that was associated with a described major repeat titer threshold. Protection levels dropped upon rechallenge 9 weeks post-1st challenge, which also corresponded to the waning of major repeat titers. Collins *et al*. have reported a similar titration of RTS,S doses in the Balb/c mouse strain, but showed saturating protection against transgenic sporozoite challenge, even at ultra-low vaccine doses^49^. Biological differences between the transgenic parasites may be one factor, but the C57Bl/6 mouse strain is generally considered harder to protect against malaria^50^. As compared to the models that rely on liver parasite burden as the readout^51^, sterile protection can be a highly stringent test to compare vaccines and monoclonal antibodies^52^. Our model clearly discerned the superiority of the particle-based RTS,S antigen over a soluble FL-CSP antigen. An ongoing Phase 2 efficacy trial with the FL-CSP will determine how mouse efficacy data would translate to CHMI^53^ (manuscript in preparation).

The RTS,S antigen was either titrated at a constant AS01 volume (AT) or proportionally titrated with varying AS01 volumes (VT). While adjuvant volume was not independently varied, the VT regimen contained lower AS01 adjuvant dose than AT. Immunogenicity and protection in AT groups saturated at a high antigen dose, but a strong antigen dose-sparing effect was most apparent at lower antigen doses. Immunogenicity and protection titrated better in the VT dose groups, resulting in ~3-fold higher PD_50_ than AT. It is possible that the differences between AT and VT dose effects are an artifact of the scaling factor between mice and humans. Previously, 1/250^th^ of a human dose of AS01 was shown to be as effective as 1/10^th^ of the human dose for stimulating IFN-γ responses in mice^10^. If the adjuvant dose used here is too high, the observed difference in AT and VT regimens may be less relevant to humans. However, antibody boosting, sterile protection, and protection correlates in mice all suggest that these observations need to be considered while designing future CHMI trials. Our data calls for independent optimization of the stoichiometric ratio of the antigen and adjuvant for human vaccines. In a practical sense, this may reduce vaccine cost and improve product availability.

ROC analysis on ELISA titer and µg/mL showed that anti-NANPx6 concentration predicted to confer sterile protection was ~3-fold or ~2-fold lower, respectively, for VT experiments compared to AT experiments. This result suggests that antibodies induced by antigen dose-sparing AT regimens were less protective per unit in the mouse model. We show that 2nd vaccination in AT elicited saturating levels of anti-NANPx6 titer and no significant boosting post-3rd immunization. Under the tested experimental conditions, we could not discern a difference in the avidity of AT and VT group antibodies. Since the protective mechanism of antibodies against sporozoites are not clearly defined, we do not know if there were mechanistic differences between AT vs. VT regimen antibody-mediated protection. It is possible that the higher doses of the adjuvant in AT led to a large proportion of antibody-producing plasma cells at 2 weeks post-3rd vaccine being elicited by the 2nd vaccination rather than the 3rd. Mechanistically, Pallikkuth et al. have suggested that the overstimulation of high-affinity B and T cell clones produced during the 1st and 2nd immunizations by the rapid delivery of a 3rd high dose causes anergy and down-regulates protective effects^54^. The presence of high levels of major repeat-binding antibodies after the 2nd dose in AT has been purported to inhibit affinity matured major repeat antibody induction by the 3rd immunization via a feedback loop^55^. In humans, a lower adjuvant dose (particularly for the 2nd vaccination) may improve boosting following 3rd and 4th vaccinations^19^. Future studies in mice need to focus on understanding how CSP-specific CD4 + T cells^56^, T follicular helper cells, dendritic cells, and the functionality of antibodies can be altered by varying immunization regimens.

While there are significant differences in protection outcomes observed in mice and humans, several clinical studies have shown promising efficacy results using reduced doses. Fractionating the RTS,S/AS01 3rd dose improved protection without increasing antibody titers^22^. Despite lower titers, RTS,S at a half dose combined with AS01_E_ showed no drop in efficacy compared to higher dose groups in CHMI conducted 3 months after the last vaccination^23^. In addition, the R21/Matrix-M pediatric trial showed that protection at lower Matrix M adjuvant dose was not significantly different from a high adjuvant dose group in a 6-month follow-up, even though titers at the lower adjuvant dose were about half those of the higher dose. Despite evidence suggesting the benefits of reducing the vaccine dose, our data also suggests that improving vaccine efficacy is more complex than simply reducing the vaccine dose. For example, the highest protection outcome was achieved in groups containing higher antigen and adjuvant doses, even within the VT groups. Despite a lower protective antibody threshold in VT experiments, AT experiments had a 3-fold lower PD_50_, suggesting the need to balance antibody quality with vaccine potency for CSP vaccines under development^57,58^. RTS,S/AS01 is the only recommended vaccine for protection against *P. falciparum* malaria in humans, and establishing its efficacy as a benchmark in a mouse model of vaccine-mediated protection will be useful for down-selecting improved malaria vaccines in the future. Establishing the PD_50_ vaccine dose and protective antibody threshold titer can act as a starting point for future mouse studies aimed at improving upon the current CSP-based vaccines.

## Methods

### Ethics statement

Animal procedures were conducted in compliance with the Animal Welfare Act and other federal statutes and regulations relating to animals and experiments involving animals and adhere to principles stated in the Guide for the Care and Use of Laboratory Animals, NRC Publication, 2011 edition. Studies involving animals were performed according to an IACUC-approved protocol.

### Mice and immunizations

C57Bl/6 mice were purchased from Charles River Laboratories (Holister, CA). RTS,S, and AS01 were supplied by GSK, and stored at −80 °C and 4 °C, respectively. 50 µL of AS01 contained 2.5 µg of 3-O-desacyl-4′-monophosphoryl lipid A (MPL) and 2.5 µg of *Quillaja saponaria* Molina, fraction 21 (QS-21) and liposome. Soluble FL-CSP was a recombinant nearly full-length *P. falciparum* circumsporozoite protein (containing the C- and N-terminus regions, with 19 NANP and 3 NVDP repeats in the repeat region) and was produced using good manufacturing practice^40,59^. RTS,S was formulated with the adjuvant per instructions provided by GSK. Formulations of FL-CSP in AS01 contained 0.75 µg CSP per 25 µL and was mixed 1:1 with a 2x stock of AS01. All immunizations were administered intramuscularly as a 50 µL volume in the outer left thigh at three-week intervals.

### Challenge

For assessment of vaccination-induced protection against malaria, mice were challenged 2 weeks post-3rd immunization by a 100 µL intravenous injection through the tail vein with 3000 transgenic *P. berghei* sporozoites expressing *P. falciparum* CSP^39^. Mice were monitored daily from days 4–15 for parasitemia by blood smear. Mice that showed parasitemia on 2 consecutive days were considered non-protected and euthanized. Nine weeks after the initial challenge (11 weeks after the 3rd dose), the surviving mice were rechallenged in the same manner.

### Bleeds and ELISA

Each mouse was bled 3 weeks after the 1st and 2nd immunizations and 2 weeks after the 3rd immunization. Sera were used to analyze antibody titers by ELISA. Full-length CSP (100 ng/well), NANPx6-C peptide (100 ng/well), Pf16 a biotinylated C-terminal peptide (EPSDKHIKEYLNKIQNSLSTEWSPCSVTCGNGIQVRIKPGSANKPKDELDYANDIEKKICKMEKCS [100 ng/well]), or purified Hepatitis B *S* antigen supplied by GSK (100 ng/well) in PBS were coated on Immulon 2HB 96-well microtiter plates (Thermo Scientific, Rochester, NY). For ELISA^59^, plates were coated overnight at 4 °C and blocked the next day for 1 h with 1% casein in PBS. Serum was serially diluted and added to the plate for 2 h at room temperature. Secondary antibody anti-mouse IgG HRP (Southern Biotech, Birmingham, AL) was then added for 1 h. Plates were developed with ABTS peroxidase substrate system (KPL, Gaithersburg, MD) for 1 h, and the reaction was stopped with a final 2.5% concentration of sodium dodecyl sulfate. Washes between each step were performed with PBS + 0.05% Tween20. Titer was calculated as the dilution that resulted in OD_415_ = 1.0 using Gen5 4-parameter nonlinear regression (BioTek, Winooski, VT).

### Avidity assay

Duplicate plates were coated with 100 ng/ml NANPx6 peptide were blocked using 0.5% casein and 1% Tween-20. Sera were diluted 1:500 and 100 µL aliquots per well were diluted 3-fold down the plate, following 2 h incubation one of the plates was washed with PBS and the other with 1 M sodium thiocyanate for 15 minutes. The remaining ELISA was developed as above. Titers were calculated as the dilution that resulted in OD_415_ = 1.0 using Gen5 4-parameter nonlinear regression (BioTek, Winooski, VT) and avidity index was calculated as the ratio of OD = 1 titers for the sodium thiocyanate and PBS washed plates.

### BLI assay

Streptavidin-coated biosensors loaded with 0.45 µg/mL of NANPx6 peptide were used to obtain a standard curve (nm shift vs. µg/mL) using an equimolar mixture of 7 human repeat region monoclonal antibodies 317, 311 CIS43, MGG4, 663, 580 and 1210^52^. The assay was run on an Octet Red96 instrument (Sartorius, Freemont CA). Mouse sera were diluted to 1:50 in kinetics buffer (PBS, pH 7.5, 0.002% Tween-20 and 0.01% BSA) and biosensors were set to baseline for 120 s, load for 180 s, associate for 300 s, and dissociate for 300 s. Data were analyzed using ForteBio data analysis software HT Version 12.0. Sensorgrams were normalized to pooled naïve serum control run on each plate via signal subtraction.

### Statistical analysis

Both descriptive and statistical inference analyses were applied during the study. Comparisons of titer means were performed using ANOVA (multiple level) with Tukey’s multiple comparison applied for the continuous outcomes. Comparison of titers in two groups were performed using unpaired Mann-Whitney U test. Statistically significant differences in group means were indicated in figures as * for *P* value < 0.05, ** for *P* value < 0.01, *** for *P* value < 0.001, and **** for *P* value < 0.0001. For the sterile protection outcomes, contingency tables were analyzed for the binary outcome and Fisher’s exact test was used to assess the homogeneity. To examine the performance of antibody titer outcomes to predict protection, 2 weeks post-3rd vaccination titers were analyzed using Receiver Operating Characteristic (ROC) curves. Area under the Curve (AUC) was used as the evaluation metric to check the classification performance. The shortest distance between the ROC curve and the theoretical “100% sensitivity and 100% specificity” point were used to determine an ideal cut-off titer for protection outcomes. A two parameter log logistic model was used to estimate the PD_50_ dose of the antigen (relative potencies using dose vs. protection response between AT and VT groups). Modeling was performed using the following relationship using the EZAnalytics website (https://ce.ezanalytix.com; London, UK) and SAS 9.4 was used for analysis. x= dose; b= slope; e = intercept (PD_50_).

$$f(x)=\frac{1}{1+{\exp}\left(\right.b({\log}(x)-{{\log}}(e))}$$

In another analysis individual mouse titers were separated into bins of incremental antibody concentrations (bin size of 25 µg/mL or 0.33 log titer) and protection for each bin was calculated. The best fitting dose–response curve between antibody titer and percentage protection was modeled according to the Hill equation using GraphPad Prism 9.

### Reporting summary

Further information on research design is available in the Nature Research Reporting Summary linked to this article.

### Supplementary information

Supplementary Materials

Reporting Summary

## Data Availability

Raw data collected during this study can be made available upon request.
