# Supplementary material for: Protective antibody threshold of RTS,S/AS01 malaria vaccine correlates antigen and adjuvant dose in mouse model
Source: NPJ Vaccines. 2023 Aug 10;8:114. doi: 10.1038/s41541-023-00714-x (PMC10415390; doi:10.1038/s41541-023-00714-x)
Supplement: Supplementary file 1 — Supplementary Materials [file 41541_2023_714_MOESM1_ESM.pdf]

SUPPLEMENTARY MATERIAL

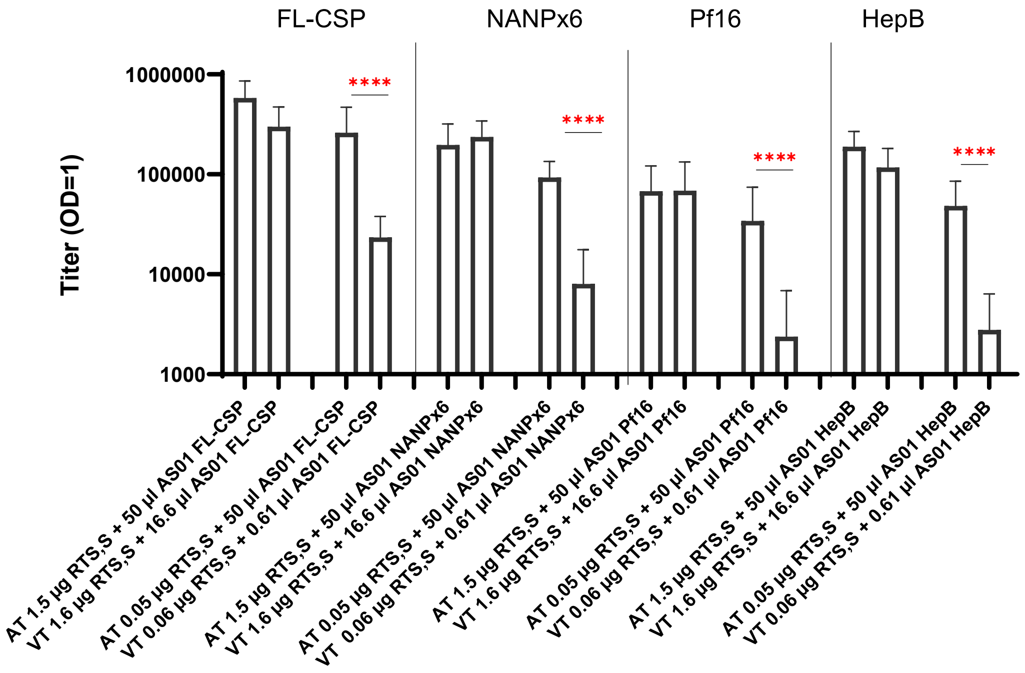

Supplementary Figure 1: Immunogenicity of AT and VT groups at similar antigen doses. GMT (± 95% CI) against FL-CSP, NANPx6, Pf16, and HepB at 2 weeks post-3rd vaccine for AT 1.5 µg RTS,S + 50 µL AS01 *vs.* VT 1.6 µg RTS,S + 16.6 µL AS01 or AT 0.05 µg RTS,S + 50 µL AS01 *vs.* VT 0.06 µg RTS,S + 0.61 µL AS01. Significant differences in between group mean were determined by Mann-Whitney test; ****P<0.0001.

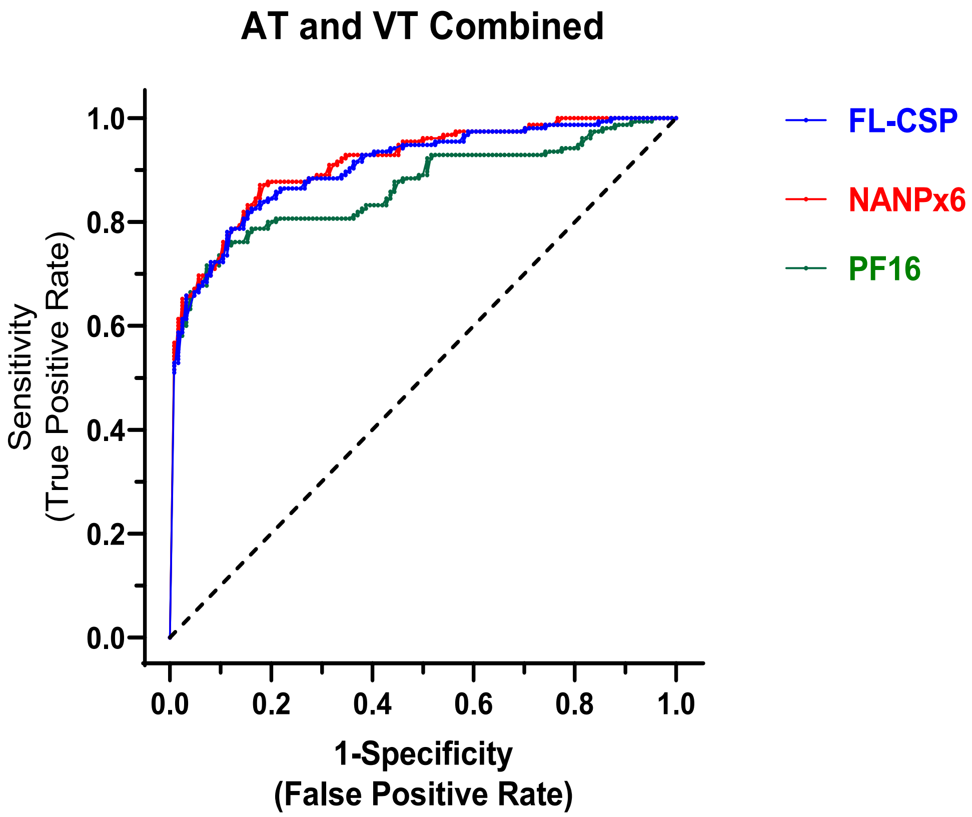

Supplementary Figure 2: Receiver operator characteristic (ROC) curve. Sensitivity *vs.* 1-Specificity for a range of 2 weeks post-3rd vaccine FL-CSP, NANPx6, and Pf16 ELISA titers were plotted for AT and VT studies combined. Dashed line denotes the random guessing line. The shortest distance between the ROC curve and a theoretical "100% sensitivity and 100% specificity" point (rho factor) was used to determine the cut-off titer for optimal prediction of challenge outcome.

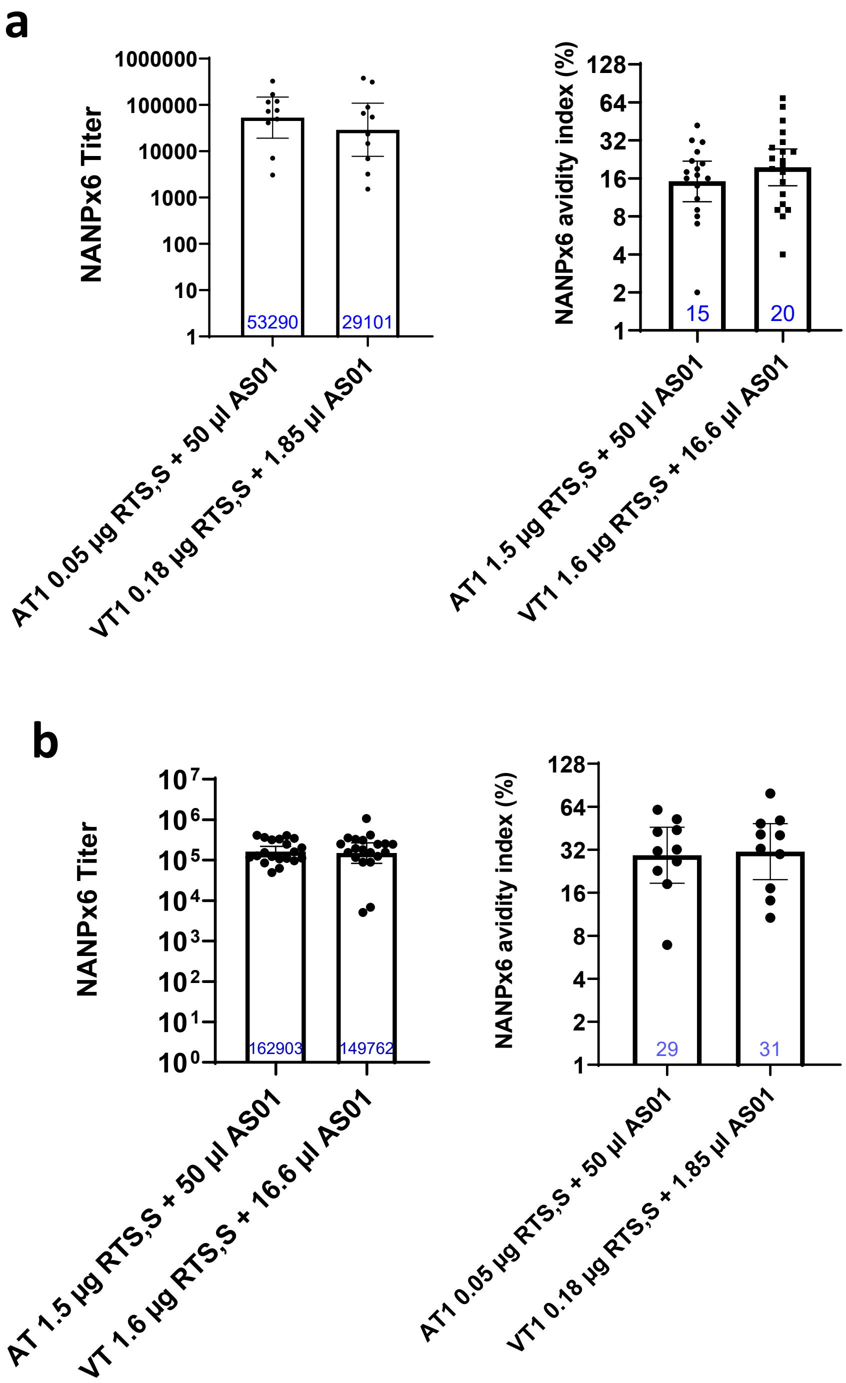

Supplementary 3: Avidity index AT *vs.* VT. Avidity was measured using NANPx6 ELISA on individual mice at 2 week post 3rd vaccine in groups that received, **(a)** similar antigen doses; or **(b)** elicited similar ~50% protection.

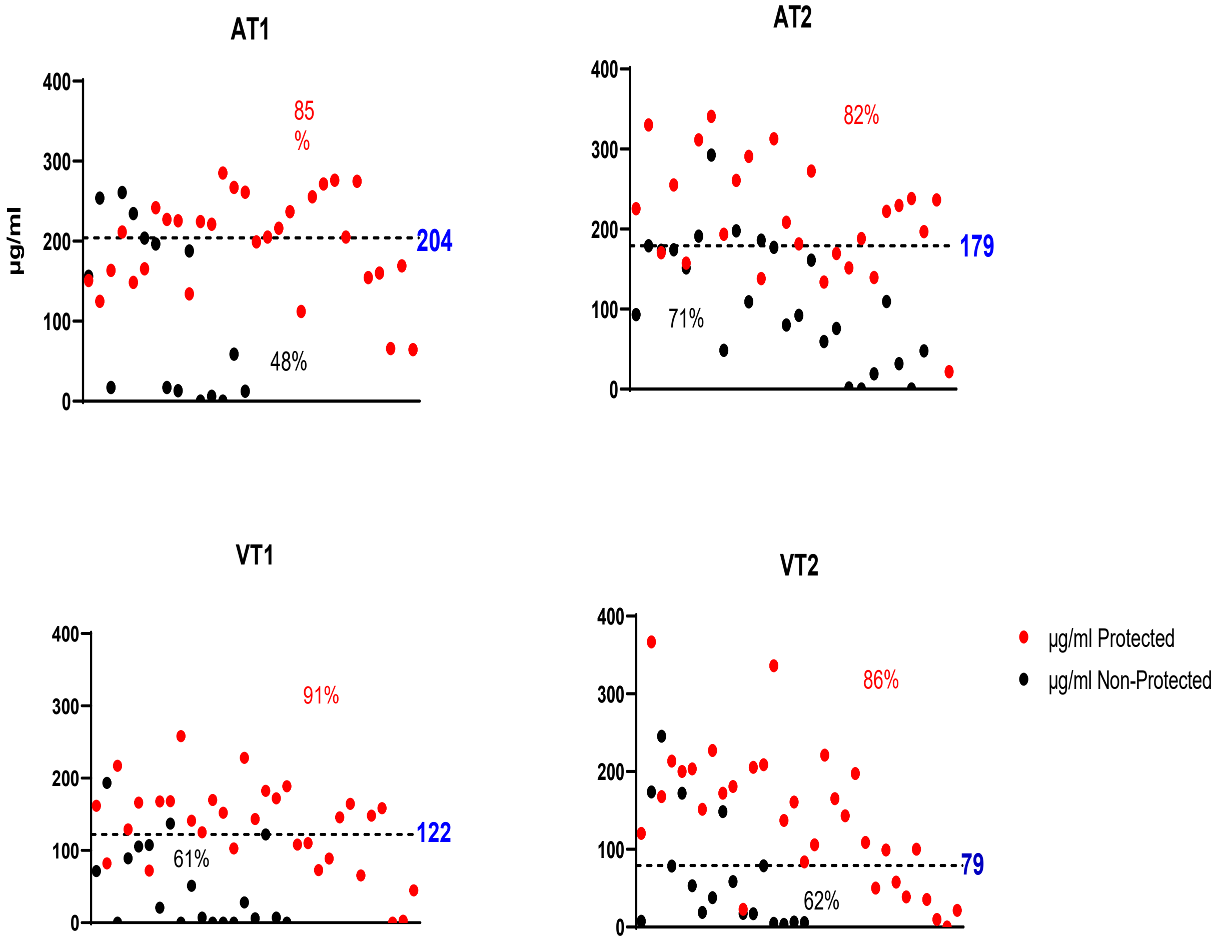

Supplementary 4: BLI µg/mL major repeat antibody concentrations in AT1, AT2, VT1, and VT2 mouse studies. The dotted line denotes the titer cut-offs (blue) predicted by ROC analysis. The percentage of mice protected (red) above the cutoff and percentage of mice infected (black) below the predicted cutoff are shown.
