# Supplementary material for: Protective antibody threshold of RTS,S/AS01 malaria vaccine correlates antigen and adjuvant dose in mouse model
Source: NPJ Vaccines. 2023 Aug 10;8:114. doi: 10.1038/s41541-023-00714-x (PMC10415390; doi:10.1038/s41541-023-00714-x)
Supplement: Supplementary file 2 — Reporting Summary [file 41541_2023_714_MOESM2_ESM.pdf]

nature portfolio

Corresponding author(s): Sheetij Dutta

Last updated by author(s): Jul 7, 2023

Reporting Summary

Nature Portfolio wishes to improve the reproducibility of the work that we publish. This form provides structure for consistency and transparency in reporting. For further information on Nature Portfolio policies, see our Editorial Policies and the Editorial Policy Checklist.

Statistics

For all statistical analyses, confirm that the following items are present in the figure legend, table legend, main text, or Methods section.

n/a	Confirmed	
☐	☒	The exact sample size (*n*) for each experimental group/condition, given as a discrete number and unit of measurement
☐	☒	A statement on whether measurements were taken from distinct samples or whether the same sample was measured repeatedly
☐	☒	The statistical test(s) used AND whether they are one- or two-sided<br>*Only common tests should be described solely by name; describe more complex techniques in the Methods section.*
☐	☒	A description of all covariates tested
☐	☒	A description of any assumptions or corrections, such as tests of normality and adjustment for multiple comparisons
☐	☒	A full description of the statistical parameters including central tendency (e.g. means) or other basic estimates (e.g. regression coefficient) AND variation (e.g. standard deviation) or associated estimates of uncertainty (e.g. confidence intervals)
☐	☒	For null hypothesis testing, the test statistic (e.g. *F*, *t*, *r*) with confidence intervals, effect sizes, degrees of freedom and *P* value noted<br>*Give P values as exact values whenever suitable.*
☒	☐	For Bayesian analysis, information on the choice of priors and Markov chain Monte Carlo settings
☒	☐	For hierarchical and complex designs, identification of the appropriate level for tests and full reporting of outcomes
☒	☐	Estimates of effect sizes (e.g. Cohen's *d*, Pearson's *r*), indicating how they were calculated

Our web collection on statistics for biologists contains articles on many of the points above.

Software and code

Policy information about availability of computer code

Data collection	Gen5 (BioTek, Winooski, VT) for ELISA optical density readings
Data analysis	Gen5 (BioTek, Winooski, VT) for ELISA titer calculations, ForteBio data analysis software HT Version 12.0 for BLI assay, statistics and modeling were performed using Graphpad Prism 9, SAS 9.4, and EZAnalytics website (https://ce.ezanalytix.com; London, UK)

For manuscripts utilizing custom algorithms or software that are central to the research but not yet described in published literature, software must be made available to editors and reviewers. We strongly encourage code deposition in a community repository (e.g. GitHub). See the Nature Portfolio guidelines for submitting code & software for further information.

Data

Policy information about availability of data

All manuscripts must include a data availability statement. This statement should provide the following information, where applicable:

- Accession codes, unique identifiers, or web links for publicly available datasets
- A description of any restrictions on data availability
- For clinical datasets or third party data, please ensure that the statement adheres to our policy

Data collected during this study will be made available upon request.

Research involving human participants, their data, or biological material

Policy information about studies with human participants or human data. See also policy information about sex, gender (identity/presentation), and sexual orientation and race, ethnicity and racism.

Reporting on sex and gender	This manuscript does not contain research involving human participants, data, or biological material.
Reporting on race, ethnicity, or other socially relevant groupings	*Please specify the socially constructed or socially relevant categorization variable(s) used in your manuscript and explain why they were used. Please note that such variables should not be used as proxies for other socially constructed/relevant variables (for example, race or ethnicity should not be used as a proxy for socioeconomic status).*<br>*Provide clear definitions of the relevant terms used, how they were provided (by the participants/respondents, the researchers, or third parties), and the method(s) used to classify people into the different categories (e.g. self-report, census or administrative data, social media data, etc.)*<br>*Please provide details about how you controlled for confounding variables in your analyses.*
Population characteristics	*Describe the covariate-relevant population characteristics of the human research participants (e.g. age, genotypic information, past and current diagnosis and treatment categories). If you filled out the behavioural & social sciences study design questions and have nothing to add here, write "See above."*
Recruitment	*Describe how participants were recruited. Outline any potential self-selection bias or other biases that may be present and how these are likely to impact results.*
Ethics oversight	*Identify the organization(s) that approved the study protocol.*

Note that full information on the approval of the study protocol must also be provided in the manuscript.

Field-specific reporting

Please select the one below that is the best fit for your research. If you are not sure, read the appropriate sections before making your selection.

☒ Life sciences ☐ Behavioural & social sciences ☐ Ecological, evolutionary & environmental sciences

For a reference copy of the document with all sections, see nature.com/documents/nr-reporting-summary-flat.pdf

Life sciences study design

All studies must disclose on these points even when the disclosure is negative.

Sample size	The initial pilot study established that a group size of n=10 was sufficient to determine at least a half-log difference in NANP titers between groups with statistical power >0.8.
Data exclusions	No data was excluded from the study.
Replication	Each replicated mouse study is included in the manuscript and similar results were obtained between trials.
Randomization	Mice were arbitrarily assigned to experimental groups. Sex and age were matched between groups in the same experiment.
Blinding	Data collection was not blinded.

Reporting for specific materials, systems and methods

We require information from authors about some types of materials, experimental systems and methods used in many studies. Here, indicate whether each material, system or method listed is relevant to your study. If you are not sure if a list item applies to your research, read the appropriate section before selecting a response.

Materials & experimental systems

n/a	Involved in the study	
☐	☒	Antibodies
☒	☐	Eukaryotic cell lines
☒	☐	Palaeontology and archaeology
☐	☒	Animals and other organisms
☒	☐	Clinical data
☒	☐	Dual use research of concern
☒	☐	Plants

Methods

n/a	Involved in the study	
☒	☐	ChIP-seq
☒	☐	Flow cytometry
☒	☐	MRI-based neuroimaging

Antibodies

Antibodies used	HRP-conjugated goat anti-mouse IgG (polyclonal, SouthernBiotech cat# 1036-05), human IgG1 monoclonal antibodies against the CSP repeat region (317, 311 CIS43, MGG4, 663, 580, and 1210) were made at WRAIR.
Validation	Anti-mouse IgG was quality tested for ELISA by the manufacturer (SouthernBiotech); human monoclonal antibodies were tested for specificity to the CSP repeat region by Western blot and ELISA (Livingstone et al., Sci Rep 2021 doi: 10.1038/s41598-021-84622-x).

Animals and other research organisms

Policy information about studies involving animals; ARRIVE guidelines recommended for reporting animal research, and Sex and Gender in Research

Laboratory animals	C57Bl/6 mice were purchased from Charles River Laboratories at 6-8 weeks of age.
Wild animals	This study did not involve wild animals.
Reporting on sex	This study used female mice to remain consistent with previous benchmark testing of vaccine efficacy in the WRAIR mouse model.
Field-collected samples	This study did not include samples obtained from the field.
Ethics oversight	Animal procedures were conducted in compliance with the Animal Welfare Act and other federal statutes and regulations relating to animals and experiments involving animals and adhere to principles stated in the Guide for the Care and Use of Laboratory Animals, NRC Publication, 2011 edition. Studies involving animals were performed according to an IACUC-approved protocol.

Note that full information on the approval of the study protocol must also be provided in the manuscript.
